# Frame-based mathematical models – a tool for the study of molecular genetic systems

**DOI:** 10.18699/vjgb-25-135

**Published:** 2025-12

**Authors:** F.V. Kazantsev, S.A. Lashin, Yu.G. Matushkin

**Affiliations:** Institute of Cytology and Genetics of the Siberian Branch of the Russian Academy of Sciences, Novosibirsk, Russia Kurchatov Genomic Center of ICG SB RAS, Novosibirsk, Russia Novosibirsk State University, Novosibirsk, Russia; Institute of Cytology and Genetics of the Siberian Branch of the Russian Academy of Sciences, Novosibirsk, Russia Kurchatov Genomic Center of ICG SB RAS, Novosibirsk, Russia Novosibirsk State University, Novosibirsk, Russia; Institute of Cytology and Genetics of the Siberian Branch of the Russian Academy of Sciences, Novosibirsk, Russia Kurchatov Genomic Center of ICG SB RAS, Novosibirsk, Russia

**Keywords:** Hill functions, mathematical modelling, gene networks, frame-based models, domain-specific languages, функции Хилла, математическое моделирование, генные сети, фреймовые модели, предметно-ориентированные языки спецификации модели

## Abstract

This paper reviews existing approaches for reconstructing frame-based mathematical models of molecular genetic systems from the level of genetic synthesis to models of metabolic networks. A frame-based mathematical model is a model in which the following terms are specified: formal structure, type of mathematical model for a particular biochemical process, reactants and their roles. Typically, such models are generated automatically on the basis of description of biological processes in terms of domain-specific languages. For molecular genetic systems, these languages use constructions familiar to a wide range of biologists in the form of a list of biochemical reactions. They rely on the concepts of elementary subsystems, where complex models are assembled from small block units (“frames”). In this paper, we have shown an example with the generation of a classical repressilator model consisting of three genes that mutually inhibit each other’s synthesis. We have given it in three different versions of the graphic standard, its characteristic mathematical interpretation and variants of its numerical calculation. We have shown that even at the level of frame models it is possible to identify qualitatively new behaviour of the model through the introduction of just one gene into the model structure. This change provides a way to control the modes of behaviour of the model through changing the concentrations of reactants. The frame-based approach opens the way to generate models of cells, tissues, organs, organisms and communities through frame-based model generation tools that specify structure, roles of modelled reactants using domain-specific languages and graphical methods of model specification.

## Abstract (ru)

Представлен обзор подходов реконструкции фреймовых математических моделей молекулярно-генетических систем от уровня генетического синтеза до метаболических сетей. Фреймовые математические модели – это модели, в которых заданы: формальная структура, математический формализм под конкретный биохимический процесс, реагенты этого процесса и их роль в нем. Обычно такие модели созданы в автоматическом режиме на базе описания биологического процесса в терминах языков предметной области. Для молекулярно-генетических систем такие языки используют конструкции, привычные для широкого круга исследователей-биологов, например список биохимических реакций. Они основываются на концепции элементарных подсистем, где комплексная модель собрана из небольших блоков – «фреймов». В настоящей работе мы показали пример с генерацией классической модели репрессилятора, состоящей из трех генов, продукты синтеза которых последовательно ингибируют биосинтез друг друга. Для этого примера мы привели три версии графической нотации описания структуры модели, их типичную математическую интерпретацию и варианты вычислительных экспериментов. Показали, что даже на уровне фреймовых моделей возможно идентифицировать качественно новое поведение благодаря добавлению в структуру модели всего одного гена. Такая модификация предоставляет пути контроля режимами функционирования модели через изменение концентраций ее реагентов. Подход, основанный на фреймах, открывает пути генерации моделей клеток, тканей, органов, организмов и сообществ с помощью программных инструментов, которые формализуют структуру модели и роль ее реагентов, используя как предметно-ориентированные языки, так и графические методы спецификации моделей

## Introduction

In the era of accumulation of large genetic data, the question of high-throughput analysis of these data using methods of mathematical and computer modelling has arisen. The last 30 years of scientific experience have prepared the theoretical basis for computational analysis tasks. The mechanisms of biochemical catalysis were studied, the rates of biochemical processes were estimated, and the scenarios of transcriptional synthesis and the influence of external factors were examined (Alon, 2007; Wittig et al., 2018; Kolmykov et al., 2021; Vorontsov et al., 2024; Rigden, Fernández, 2025). A mathematical foundation has been prepared, which, together with the development of experimental and computational technologies, has set the trend for the transition from small models of enzymatic kinetics to full-genome models of bacterial (Karr et al., 2014) and animal cells (Norsigian et al., 2019). Existing approaches rely on the concepts of elementary subsystems, where complex models are assembled from small block units. The rejection of “monolithic” models in a single mathematical formalism (e. g. the ODE system) in favour of representing and storing models as a set of model units has set the direction for the integration of multiple tools through standards. These model units carry structural information about the role of each reactant and the mathematical interpretation of the biological process (Malik-Sheriff et al., 2019). This tremendous amount of knowledge allows to pass through ordering of information to the formalization of models, that is, the ability to propose a formal structure and type of mathematical model for a specific biochemical process with known reactants – a frame model. If we can offer a formal algorithm for translating knowledge into a model, then the way of setting the initial configuration can be left as usual for scientists – through a domain-specific language to describe the model

## What are frame models?

Frame-based mathematical models in the field of molecular genetic systems (MGS) modelling are most often understood as models that have been made using domain-specific model description languages (DSL) and tools for model generation on their basis. For MGS, such languages use constructions familiar to a wide range of biologists in the form of a list of biochemical reactions, for example (Shapiro et al., 2003, 2013; Hoops et al., 2006). This way allows one to set the reactants of biochemical processes and their roles. It is the information about the role of reactants that is the necessary element for algorithms of frame models construction, crucial parameters for formal generation rules. Moreover, the mathematical formalism may differ depending on the problem to be solved. These can be likened to models in the form of ODE system, discrete models or based on Boolean logic (Beal et al., 2011; Galdzicki et al., 2014).

In this paper, we review approaches for describing models that consider only the levels of transcription, translation, enzymatic synthesis, signaling networks and metabolic pathways. This limitation reveals the structure and hierarchical arrangement of such models, from the basic processes of synthesis to the arrangement of everything into a system of interacting metabolic pathways. Within the framework of building such MGS models, the bottom-up approach is natural – when one moves from models of simple processes to their combination, obtaining a synergistic/emergent effect (Kolodkin et al., 2012, 2013). This process of increasing the model complexity resembles a design based on the principle of staking “domino tiles” by the rule of reactants overlapping

In addition to domain specific languages, the process of designing frame models can be started from a structural model/ schema/graph of interacting entities with specified roles of participants. Such a graphical way of specifying the reactants and their roles for modelling is more illustrative and allows the use of additional analysis tools in case of working with a big amount of data. These graphs are the input markup for the frame model generation stage. In general, it is possible to unambiguously switch from representing the role of reactants from a list of biochemical reactions to a graph representation and vice versa. There are several standards for the presentation of structural information: SBML (Hucka et al., 2015), SBGN (Moodie et al., 2015), SBOL (Galdzicki et al., 2014).

## Basic theorems underlying the approaches

Some theoretical issues concerning the integration of frame models are worth clarifying first. The simple frame models of MGS are built on the basis of chemical kinetics equations representing various ways of the kinetic mass action law. The construction is carried out within the paradigm of local independence of functional properties of elementary subsystems from their compartmental localization within the original system. If a particular structural model or reactions scheme is available, the instantaneous concentration rate of any substance is equal to the sum of the local concentration rates of that substance for each reaction in which that substance participates as a substrate or product. The theoretical basis for this is Korzukhin’s theorem, which is crucial for modelling chemical kinetics and states: “For any set of non-negative curves given on a finite time interval and any given accuracy, there exists such (there may be more than one) biochemical scheme composed only of bimolecular and monomolecular reactions that the mathematical model constructed from this biochemical scheme approximates the given set of curves with a given accuracy” (Zhabotinsky, Zaikin, 1973). The extension of these ideas is formulated in the framework of the generalised chemical kinetic modelling method (GCKMM), proposed by Vitaly Likhoshvay (Likhoshvai et al., 2001). Some examples of this approach are presented below.

## Frame model examples

**Processes of genetic synthesis – transcription, translation**

**Fig. 1.**
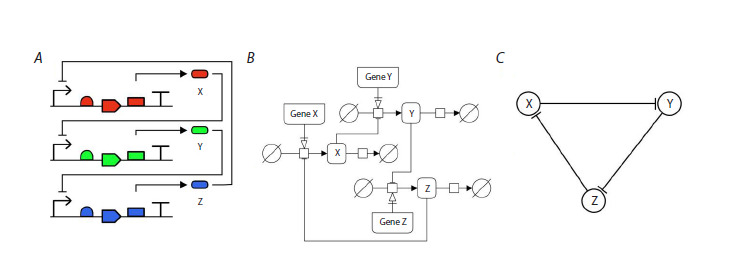
Graphical representation of the repressilator model in different standards: A, SBOL notation describes in detail the arrangement/ structure of a DNA molecule and the functional relationship between the elements. B, SBGN notation carries more information about the processes that compose the model. C, Notation of protein-protein regulatory interactions, specific for describing Hypothetical Gene Networks (omits many details of the mechanisms).

When designing frame models at the level of genetic synthesis, it is necessary to set the process structure and roles of the reactants in the process. These reactants are genes and transcription factors. An interesting approach to describe the genetic level is the SBOL approach (Galdzicki et al., 2014) (The Synthetic Biology Open Language, sbolstandard.org). It allows describing the structure of a DNA molecule with the location of genes, binding sites of transcription factors, regulatory relations of synthesis products from genes and some other properties. It is possible to describe them both in text form using a domain-specific modelling language or in a graph form using a special graphical editor (Der et al., 2017; Cox et al., 2018). There is a set of tools for working with this standard and tools for graphical interpretation of such models (Fig. 1A).

For further presentation a demonstration of a well-known three genes model is given, each of them inhibits synthesis from a neighboring gene – a repressilator (Elowitz, Leibler, 2000). Figure 1 shows a possible graphical interpretation of such a model under various types of representation

**Fig. 2.**
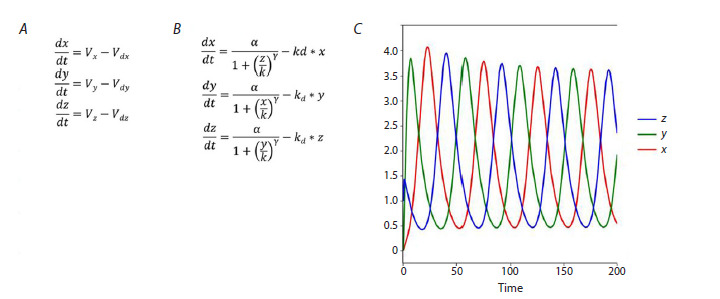
Generalized view of the frame mathematical model and characteristic plots of concentration variation with time obtained for the structure from Figure 1. A, Model representation as combination of elementary subsystems. Vi – as synthesis processes, Vdi – as dissipation processes. B, The model ODE system description. C, The dynamics is obtained with parameters γ > 2, α = 1, k = 0.5, kd = 0.1 and initial point x0 = y0 = 0, z0 = 1.0.

Once the structure of relations between molecules is given, it is possible to build a “frame mathematical model” describing the dynamics of the process. The role of the element allows one to understand at a glance the contribution of the selected subsystem and its negative/positive effect in the overall system of equations (Fig. 2A). For this purpose, we can use approaches that are described in such tools as SBMLSqeezer (Dräger et al., 2015), MGSgenerator (Kazantsev et al., 2009), Micro- TranscriptMod (Lakhova et al., 2022). These tools, given the role of each of the reactants in the process, generate equations for the rate of product synthesis (Fig. 2B). The more details on the role of the reactants are described, the more precisely their behavior can be specified in the model. At the stage of computational experiments, all such tools combine elementary subsystems (separate processes) into one unifying structure, the one common model. The typical view of the mathematical model and its dynamics for each of the structures presented in Figure 1 will look as it is shown in Figure 2.

**Hypothetical gene networks**

**Fig. 3.**
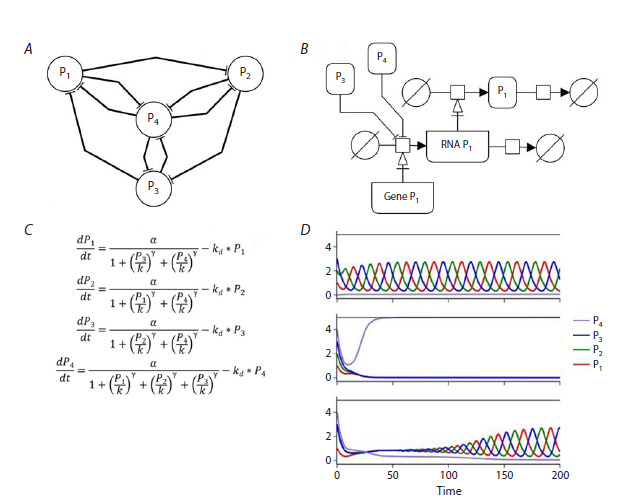
A four-node model of hypothetical gene network (HGS) and its characteristic behavior. A, Structural model, where arcs define the conditions of biosynthesis inhibition. B, An extended description of the processes behind each of the HGS nodes in SBGN standard. C, Model equations that correspond to the structure. D, Characteristic plots of concentration vs time obtained for the presented structure from Figure A. The dynamics are obtained at parameters γ = 3, α = 1, kb = 0.5, kd = 0.2 and starting points [P1, P2, P3, P4]: (1) [1.0, 2.0, 3.0, 0.0]; (2) [1.0, 2.0, 3.0, 4.0]; (3) [1.0, 2.8, 3.0, 4.0].

A model does not often require excessive detail. There is a class of models where the relationship of genes and their synthesis products with other genes is modelled. The structural model is represented by a unipartite graph, where each node represents both the process of transcription of the coding part of a gene and translation of its mRNA (or synthesis of its protein) (Fig. 1С, Fig. 3). A graph node is considered as a unified transcription-translation process. Directed arcs (arrows) in such a graph define an inhibiting or activating effect on another node (on itself is also allowed). This class of models is named Hypothetical Gene Network (HGN) (Likhoshvai et al., 2003).

Hypothetical gene networks with cyclic inhibitory effects of reactants (which are specified with the relationships: “protein Pi inhibits the synthesis of product Pj from gene gj”, see Figure 3) are exhaustively described in (Fadeev, Likhoshvai, 2003). Each edge in a graph representation of such models affects the generalized transcription/translation process of the node to which it is directed. Moreover, when generating ODE models for these graphs, a third process – decay of the synthesis product – is added to the mentioned processes. A node in such graphs is understood as follows: “An RNA molecule ri is synthesised from the gene gi, from which a Pi protein is synthesised and this protein is degraded/dissociated over time”. Figure 3A shows an example of an HGS model of four nodes and nine edges specifying the conditions of biosynthesis inhibition. The structure is obtained by inserting one additional node into the model shown in Figure 1. The additional node inhibits the others, and they in turn inhibit it. The resulting mathematical model is presented in Figure 3C. While the model presented in Figure 1 has one unstable steady-state condition and stable oscillatory behaviour under parameters presented in Figure 2, the introduction of an additional node allows the behaviour of the model to change depending on the concentration of reactants (Figure 3D): at low initial concentrations of P4 the system oscillates stably as the initial model, and at sufficiently high concentrations of P4 it enters the stationary regime. Moreover, regime switching can be controlled by small changes in concentrations. Changing the regulation mechanism to non-competitive does not change the regimes of the model behavior. The model found in Supplementary Materials1 as a Copasi file is a model version with the non-competitive regulation mechanism in it

Supplementary Materials are available in the online version of the paper: https://vavilovj-icg.ru/download/pict-2025-29/appx50.zip


Studying such a class of models and forming a knowledge base of their dynamics allows us to identify possible behavior at the level of structural models of target biological systems without performing calculations

**Enzymatic reaction**

For enzymatic synthesis processes, the key aspect is the presence of an enzyme, which catalyzes the process but is not consumed in the course of the reaction. To reconstruct a model of enzymatic reactions, the reaction mechanisms, the order of interaction of molecular players of the reaction with the enzyme, the steps of transformation and release of the product should be taken into account. Once one has an assumption about the mechanisms of the enzyme’s relationships with substrates and products, a suitable form of representing these interactions as a graph may be suggested. There are a number of works in this area. One can use ready-made solutions for building frame models of enzymatic reactions on graphs (King, Altman, 1956; Cornish-Bowden, 1977) (www. biokin.com/tools/king-altman/). In addition, the Copasi tool has a set of predefined frame-based mathematical models for enzymatic systems (Hoops et al., 2006). These models may not only be used as examples in a case study, but also be valuable in developing and analyzing a model within the Copasi toolkit: design a set of elementary subsystems (model structure); give them a mathematical law of velocity based on frame models; get a ready system of equations; perform computational experiments in both continuous and discrete stochastic formalisms; perform computational analyses of the model to fit the parameters to the experimental data and test the robustness of the model to variation of the parameters.

SBMLsqeezer can serve as an independent source of frame models (Dräger et al., 2015). It is both a database of ready-made model variants and a tool that can match a well-annotated structure in the form of an SBML model to a suitable model variant. This tool can be embedded into the CellDesigner application (Funahashi et al., 2008). There is a ready set of equations adapted to experimental data for both enzymatic reactions and transcription-translation processes in the bacterium E. coli (Kazantsev et al., 2018). These models may serve as a training sample because they contain accompanying information about the data items on which the models were built.

**Metabolic pathways**

Frame models of metabolic pathways can also be derived from structural information in the form of graphs. It is possible to build a model through descriptions of reactions in tabular form with COBRApy (Ebrahim et al., 2013) and BIOUML (Kolpakov et al., 2022). These tools allow the construction of a whole-genome model in terms of flux balance modelling (Orth et al., 2010). But if one needs to work within continuous models, the Path2Models project (Büchel et al., 2013) may be used, in which 140,000 frame models were generated based on structural models from the KEGG database (Kanehisa, 2000). These models are available at the biomodels.net resource (Malik-Sheriff et al., 2019). This kind of automation in model building is also available as part of the Cellerator package for the Mathematica modelling environment (Shapiro et al., 2003, 2013).

**Signalling pathways**

Signalling pathways require different approaches. Within such pathways, it is necessary to take into account the change of states of one molecule and/or the formation of molecular complexes, the change of conformational states, the consideration of active centres of molecules, etc. For automating the generation of these types models, the BioNetGen resource is being developed (bionetgen.org) (Harris et al., 2016). The key feature is that a series of allowed states of molecules is described, their active centres, and the rules of interaction through the active centres. These data are specified within a domain specific language. The visualization of this kind of relationship is specified within the “SBGN:entity relations” standard (sbgn.github.io). In order to try these models, one can use the VScode development environment module (code. visualstudio.com). BioNetGen algorithms build reaction chains themselves (structural models) and propose frame models for them in the widely used SBML standard. Then one can run a series of computational experiments using both discrete methods and continuous methods in several specialized computational tools that support SBML models as input data.

## Designing and depositing of the model

When designing models using automation and autogeneration, one faces the problem of identifying the right entity in the lists of variables and parameters. If the model is formatted as a monolithic system of differential equations within the particular syntax in one of the engineering modelling environments, one has to map each of the xi of the model to the proper biology entity through reading the accompanying publications. At best, authors will name variables as short acronyms for proteins or metabolites. On the other hand, it is more difficult to come up with some general rule for naming parameters correctly. It was the transition to the representation of MGS models as a set of elementary subsystems corresponding to an independent biochemical process that allowed to solve most of the mentioned issues (Miller et al., 2010; Hucka et al., 2015). In this concept, a model is not a set of equations, but an instruction on how to assemble a target model in the target mathematical formalism from tens, hundreds and thousands of pieces of elementary subsystems distributed over compartments and perform a series of computational experiments with it. In order to end up with a development-friendly model, it is better to follow a series of recommendations for the design of such elementary blocks using the systems biology ontology (SBO) (Courtot et al., 2011). This ontology allows to associate the rate equations of processes and the parameters of these equations with the meanings that were given in classical studies

The problem of model annotation is well highlighted in a publication on the model reproducibility crisis (Tiwari et al., 2021), where the authors showed that 51 % of mathematical models published on the largest online resource (biomodels. net) are not reproducible, for various reasons. It is the frame models that partially solve the issue of both repeatability of computational experiments and reproducibility of modelling results, as the relevant toolkits contain references to the formalism, to the methods used and correctly describe the parameters with the use of ontologies. All of those questions are studied in depth due to community efforts. One of such communities is “co.mbine.org”, an initiative to coordinate the development of various standards and formats for computational models in systems biology

## Discussion

Advancement of technology has given impulse to the processes of development of artificial languages for describing models within scientific fields. We have reviewed existing solutions for designing frame-based mathematical models of molecular genetic systems. For each of them there are specific tools for representing models and performing computational experiments. The publication (Tiwari et al., 2021) proposed metrics for evaluating the resulting models in terms of readiness for reuse in new research. If one follows the proposed guidelines for incorporating annotations into a model that can be made available to modelling tools, it will enhance the possibilities of automating model processing. Model automation is an interesting route with the goal of being able to integrate off-the-shelf subsystems into comprehensive cellular, intercellular and organ level models. More and more software libraries for engineering simulation environments are becoming available where molecular genetic systems modelling approaches can already be used. Even in questions of designing industrial samples of bacterial synthesis, one comes to embrace standardization for the subsequent automation of processes. This applies to the issues of model development, their integration into the production biotechnological cycle and monitoring with updating of knowledge bases (Herold et al., 2017).

Whereas in the 2000s there were trends towards developing proprietary solutions that included a “friendly user interface”, now the trends tend towards the use of highly specialized core software for each of the stages and/or the use of specialized libraries via API calls: VScode as an editor for BioNetGen DSL; yEd or Cytoscape as tools for displaying model structure; Copasi as a general-purpose tool for computational experiments, etc.

Data analysis is also performed by general-purpose statistical processing libraries or off-the-shelf tools (dashboard) that only need to load data. Now a necessary skill for work in systems biology is proficiency in Python/R/Bash scripting languages for building pipelines and linking data between function calls of specific libraries

## Conflict of interest

The authors declare no conflict of interest.
